# Exploring the Interplay of Working Memory, Apathy, and Mood/Emotional Factors

**DOI:** 10.3390/brainsci14010078

**Published:** 2024-01-12

**Authors:** Elisa Thellung di Courtelary, Gabriele Scozia, Stefano Lasaponara, Giorgia Aguzzetti, Fabrizio Doricchi, David Conversi

**Affiliations:** Department of Psychology, La Sapienza University of Rome, 00185 Rome, Italy; elisa.thellung@gmail.com (E.T.d.C.); gabriele.scozia@uniroma1.it (G.S.); stefano.lasaponara@uniroma1.it (S.L.); giorgia.aguzzetti@hotmail.it (G.A.); fabrizio.doricchi@uniroma1.it (F.D.)

**Keywords:** mood, working memory, apathy, executive function

## Abstract

Background: Previous investigations on healthy humans showed conflicting evidence regarding the impact of mood on working memory performance. A systematic investigation of how mood affects apathy levels in healthy participants is currently missing. Methods: We administered a visuospatial (VS) and a numerical (N) n-back task to a sample of 120 healthy individuals. In these participants, using a series of questionnaires, we also evaluated apathy, mood, working memory, perceived stress, PTSD symptoms caused by the COVID-19 pandemic outbreak, and general psychiatric symptoms. Successively, we investigated their performance in the n-back task as a function of scores to these questionnaires. Results: Participants performed better in the N block than in the VS one. Their accuracy decreased as a function of the n-back difficulty. We reported no differences in working memory performance or apathy as a function of mood, stress, or PTSD symptoms. We found that phobic anxiety negatively predicted accuracy to the numerical n-back task and that subjects with greater anxiety and difficulty in regulating emotions also showed higher levels of withdrawal from the task. Conclusion: The study’s results suggest that while mood did not significantly affect working memory performance, strong associations were found between WMQ scores and working memory capabilities.

## 1. Introduction

Working memory (WM) is the cognitive process that enables us to retain and manipulate information, even when encountering new information or distractions [1]. Meanwhile, “mood” describes our enduring emotional states, which alter our sensitivity to subsequent events in a given context [2].

Numerous studies have examined the relationship between mood disorders and working memory (WM) impairment, suggesting that depressive episodes and negative mood may significantly impact cognitive performance [3].

However, the effects of mood on WM in non-clinical individuals are not yet clear, with some studies indicating positive effects of negative emotional states on visual WM precision [4,5,6], while others have found impairments [7,8]. Still, other studies have reported mixed effects depending on the type of WM [9,10] or no effect at all [11,12,13,14]. Additionally, the measure used (i.e., reaction time vs. accuracy) may affect the results [15].

Psychological stress is another important phenomenon that is closely related to negative moods and emotional states. Researchers conducted two studies to assess the effects of stress on working memory. One study [16] found that participants under stress had slower reaction times and fewer correct responses than the control group, regardless of task difficulty. However, in another study [17], stress appeared to have a negligible effect on working memory performance. Nevertheless, since the outbreak of the COVID-19 pandemic, billions of people have been exposed to unprecedented levels of psychological stress, which must be taken into account as a potentially confounding variable when considering the relationship between mood and working memory.

Finally, apathy—a lack of motivation and goal-directed behavior—relates closely to emotional states and mood. While numerous studies examine apathy in various disorders, little research has been conducted on its effects on healthy individuals, particularly concerning mood. Despite increasing interest in isolated apathy, i.e., apathy unrelated to mood disorders, there is a lack of data on how mood impacts levels of apathy in healthy people.

Pardini et al. [18] investigated whether apathy could be detected in healthy, non-depressed adults. They found that around 1.45% of non-clinical subjects exhibited significant apathy and that these individuals showed less behavioral activation than controls. However, there were no significant differences in fatigue, depression, self-efficacy, behavioral inhibition, or perceived social skills between the two groups. However, there was a significant correlation between apathy levels and quality-of-life questionnaire scores, indicating that apathy can significantly impact healthy individuals even in the absence of depression.

Despite many studies on the effects of mood on working memory, there is little data on how mood affects apathy. Therefore, this study aims to explore the relationship between psychological stress, mood, working memory capacity, and levels of apathy in healthy individuals. Specifically, we hypothesize that lower mood will negatively impact working memory performance, resulting in lower performance on working memory tasks. We also anticipate that lower mood will increase apathy levels.

## 2. Materials and Methods

### 2.1. Subjects and Data Collection

To determine the number of participants, we ran an a priori power analysis (G*Power program) [19] using a moderate effect size of f2 = 0.15. This analysis showed that 89 participants would be needed to have a power of 0.95, considering an alpha of 0.05 (two-sided) of statistical significance for multiple regression. Based on this observation, one hundred and twenty healthy volunteers (M = 28; F = 92) between 18 and 60 (mean age 27.76 years; s.d. = 10.5) participated in the study. All data were collected online using Google Modules, where participants could complete eight different questionnaires and find the URL to the behavioral experiment. All participants provided their informed consent to participate in the study. Experimental procedures were designed in accordance with the principles of the Declaration of Helsinki and were approved prior to data collection by the Local Ethical Committee of the Psychology Department from “Sapienza” University of Rome.

### 2.2. Experimental Procedure

#### 2.2.1. Questionnaires

The battery of questionnaires used in this study included eight questionnaires measuring mood, working memory levels, apathy, perceived stress, PTSD symptoms caused by the COVID-19 pandemic, psychiatric symptoms, and difficulties in emotion regulation. More specifically:

The Working Memory Questionnaire (WMQ) is a self-administered questionnaire consisting of 30 items divided in the assessment of three different domains of working memory: short-term storage, attention, and executive function. The answers were given using a Likert scale ranging from 0 (“no problem”) to 4 (“severe problem in daily life”). The questionnaire examines daily life problems linked to WM deficits: the scale is consistent (Cronbach’s α = 0.89) and sensitive (Spearman’s Rho > 0.81) to discriminate brain-injured patients from matched controls [20,21].

The self-administered version of the Apathy Evaluation Scale (AES) includes 18 items that subjects had to answer using a Likert scale from 1 (“not at all”) to 4 (“very much”) and is designed to measure the lack of motivation not related to cognitive impairment, reduced consciousness, or emotional factors. In particular, the questionnaire examines the simultaneous reductions in the behavioral, cognitive, and emotional components of goal-directed behaviors [22,23]. AES has both good internal consistency (Cronbach’s α = 0.86) and validity (all Spearman’s Rho > 0.42).

The COVID-19 PTSD Questionnaire [24] is a self-report tool that measures the post-traumatic stress disorder risk relative to the COVID-19 pandemic emergency and comprises 19 items (Cronbach’s α = 0.94). Responses were given on a 5-point Likert scale from 0 (“not at all”) to 4 (“extremely”), and the questionnaire has good convergent validity (all Spearman’s Rho > 0.39).

The Perceived Stress Scale (PSS) [25,26] is a self-report instrument classically used to assess stress and how different situations affect feelings and perceived stress. It contains 10 items that can be assessed on a Likert scale ranging from 0 (“never”) to 4 (“very often”). The PSS shows good internal consistency (Cronbach’s α ranging from 0.75 to 0.91) and validity evidence compared to health behaviors, perceived health, stressful life events, and negative effects as criterion measures.

The Profile of Mood States (POMS) [27,28] is conceived to measure an individual’s distinct mood. It comprises 65 items, and the answers were given using a Likert scale ranging from 0 (“not at all”) to 4 (“very much”). The questionnaire shows both good consistency (Cronbach’s α = 0.75) and validity (all Spearman’s Rho > 0.51)

The Patient Health Questionnaire (PHQ-9) [29,30] is a self-report tool consisting of 9 items to which the participants had to respond using a 4-point Likert scale, ranging from 0 (“never”) to 3 (“almost every day”). It is designed to measure the severity of depression, and it scores each of the nine DSM-IV criteria as “0” to “3”. It can be used to make a tentative diagnosis of depression in at-risk populations. Its internal consistency ranges from 0.85 to 0.89, and its validity is equal to 0.88.

The Difficulties in Emotion Regulation Scale (DERS) [31,32] measures emotion regulation problems and includes 36 items associated with six different subscales: non-acceptance of emotional responses, difficulty engaging in goal-directed behavior, impulse control difficulties, lack of emotional awareness, limited access to emotion regulation strategies, and lack of emotional clarity. Participants answer with a Likert scale ranging from 1 (“rarely”) to 5 (“almost always”). The DERS has high internal consistency (Cronbach’s α = 0.93) and good validity (all Spearman’s Rho > 0.34)

The Symptom Checklist-90-R (SCL-90-R) [33,34] is a self-assessment scale composed of 90 items to evaluate psychiatric symptoms. Participants had to respond on a 5-point Likert scale, 0 (“not at all”) and 4 (“very much”). The main symptom dimensions measured are somatization, obsessive-compulsive, interpersonal sensitivity, depression, anxiety, hostility, phobic anxiety, paranoid ideation, psychoticism, and additional items useful for the clinic assessment. Internal consistency of SCL-90 is good for all subscales (Cronbach’s α values ranging between 0.70 and 0.96).

#### 2.2.2. Behavioral Task

**Figure 1:**
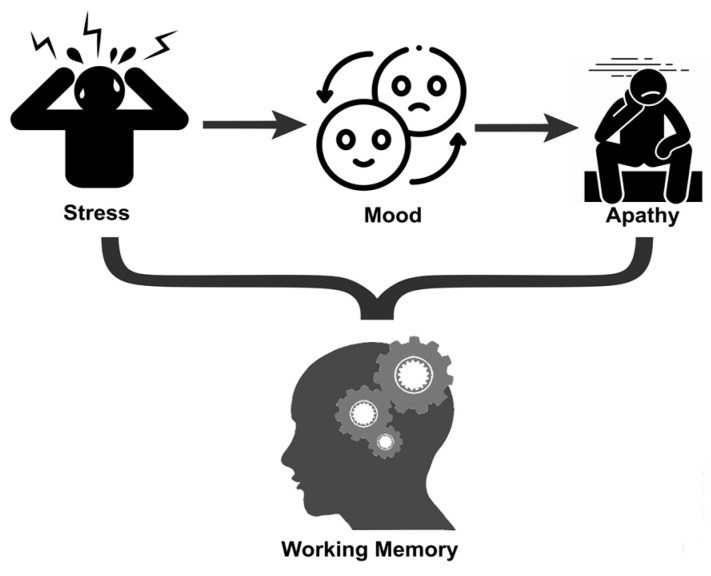
Schematic representation of the theoretical framework behind the present study.

**Figure 2:**
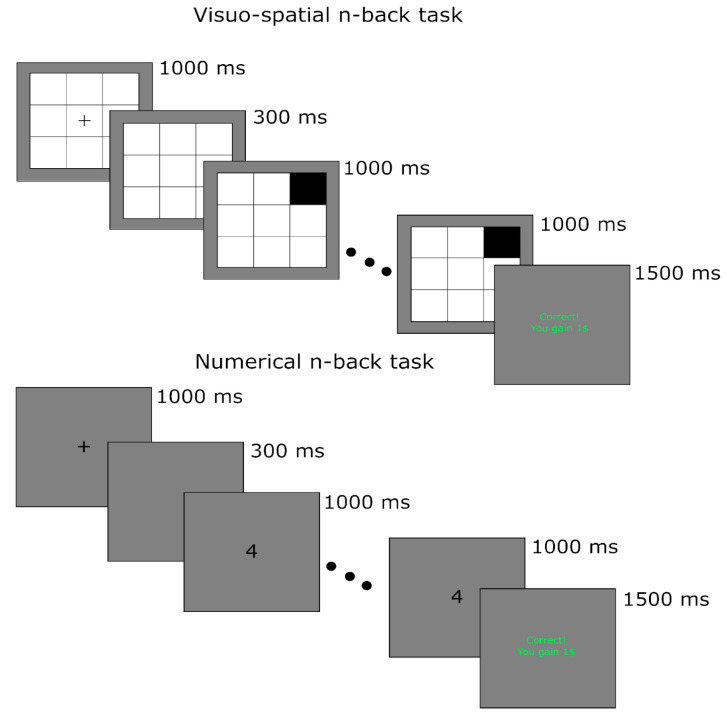
Schematic representation of visuospatial and numerical n-back task (see paragraph).

An n-back task built on the Testable platform [35] was administered to all participants (see Figure 1 and Figure 2). The n-back task is a cognitive training exercise designed to assess and improve working memory, a core component of executive functioning. It involves presenting a sequence of stimuli, typically digits or letters, and the participant’s task is to indicate whether the current stimulus matches the one presented “n” steps earlier in the sequence. The difficulty of the n-back task can be adjusted by changing the value of “n”. Higher values of n (e.g., 3-back, 4-back) require the participant to maintain a longer sequence in working memory and make more demanding comparisons [36].

Our task included two experimental parts, each divided into six blocks of trials; the first three blocks contained visuospatial stimuli (VS condition), while the second three contained digits (N condition). Both parts of the experiment involved increasing difficulty (1-back, 2-back, 3-back). In both experimental conditions, visuospatial stimuli and digits were randomly presented, controlling for at least 25 answers in each block. Each trial started with the presentation of a central fixation cross lasting 1000 ms; after 300 ms from the disappearance of central fixation, digits or spatial targets were presented for 1000 ms. Participants were requested to provide their response upon target presentation by pressing the spacebar on a computer keyboard. Following the participants’ responses, a feedback screen was presented for 1500 ms. At the beginning of the first block (1-back), participants were told that they would earn 1 virtual dollar for each correct answer; in the case of 2-back, they would earn 10 dollars for each correct answer, while during the block of 3-back, they would earn 100 dollars for each correct answer. At the end of each block, a screen with the number of correct answers appeared, informing participants about their total earnings.

To check for apathy levels, during the 2-back and 3-back blocks of both experimental parts, participants were informed that they would have the possibility to skip trials, but this would have resulted in a lower profit. Therefore, a “fake” possibility to skip a trial was randomly inserted during the task, informing participants that they could skip the subsequent trial by pressing the enter button. This “fake” possibility did not change the number of trials in experimental blocks. This likely provided a measure of apathy because skipping the subsequent trial despite a lower earning indicates a lesser interest in carrying out the task, even if this would make them earn more.

## 3. Statistical Analysis

### 3.1. Behavioral Task Performance

As a first step, participants’ performance in the n-back task was investigated as a function of the different task modalities, difficulty, and apathy level. Individual accuracies were entered in a within-subjects repeated measures ANOVA, with Modality (visuospatial vs. numerical) and Difficulty (1–2–3 back) as within factors. At the same time, the Apathy index was considered a continuous covariate. The presence of significant main effects and interactions was successively explored using Bonferroni post-hoc tests controlling for multiple comparisons.

### 3.2. Questionnaires Descriptive Statistics

Before checking for the eventual relationship between WM performance and our battery of questionnaires, we carefully explored the distribution and reliability of our scores through descriptive statistics and through Cronbach’s α calculation.

### 3.3. Relationship between Questionnaires and Behavioral Task Performance

As a second step, we checked for eventual relationships among questionnaire scores and the n-back task. In particular, this analysis allows us to assess whether and how the scores from each of our eight questionnaires can predict participants’ performance to the n-back task and the observed level of behavioral apathy, i.e., the number of times participants decided to skip a trial.

To this aim, we ran a series of stepwise linear regression analyses (see Table 1). In each model, the accuracy scores from the different conditions of the n-back task (1-backVS, 2-backVS, 3-backVS, totalVS, 1-backN, 2-backN, 3-backN, totalN), together with apathy index were separately considered as the dependent variable. The independent variable consisted of each of the eight questionnaire scales and subscales used in the study. In a further control analysis, we also considered age and sex as independent variables.

Before stepwise regression analyses, all data were carefully screened for univariate and multivariate outliers. Kolmogorov-Smirnov test for data distribution and Levene’s test for homoscedasticity were run to check for violation of the assumptions of normality and multicollinearity. Mahalanobis distance was calculated to detect and delete multivariate outliers. In all subjects, Mahalanobis distance was smaller than the critical value (all *p* > 0.001, critical value recommended by Tabachnick and Fidell) [37]. In addition, we found that variable distribution was comparable to a multivariate normal (Mardia’s multivariate kurtosis index = 160.4 ≤ 168) [38,39]. Stepwise regressions were performed with α = 0.05 using SPSS (v27). Primary associations were identified as the independent predictor with the greater association with the dependent variable (i.e., the first variable listed in the regression equation). Collinearity diagnostics were monitored with variance inflation factors < 5.0 considered acceptable. Z scores were used to stabilize the scales and improve the algorithm’s convergence for estimating the parameters of the Gaussian mixture model.

## 4. Results

### 4.1. Behavioral Task Performance

**Figure 3:**
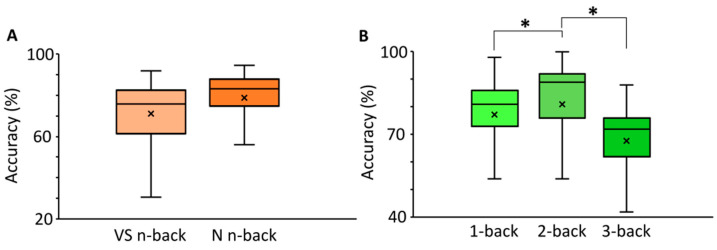
Box plots of accuracy as a function of Modality (**A**) and Difficulty (**B**) in the n-back task. Bars indicate 95% confidence intervals. * indicate a statistically significant difference at *p* < 0.05; x indicate mean values.

Participants performed better in the N block than in the VS one (Modality main effect: F (1,118) = 33.5, *p* < 0.001, ƞp2 = 0.22; see Figure 3A). Independently on the n-back modality (Difficulty main effect: F (2,236) = 38.3, *p* < 0.001, ƞp2 = 0.24), participants had a better performance in the 2-back (80%) as compared to 1-back (77%; see Figure 3B). However, in all cases, their accuracies decreased as a function of the n-back difficulty, with lower scores in the 3-back (67%; see Figure 3B). No significant main effect or interaction (all *p* > 0.21) related to the Apathy covariate was found. It should provide a concise and precise description of the experimental results, their interpretation, as well as the experimental conclusions that can be drawn.

### 4.2. Questionnaires Descriptive Statistics

**Figure 4:**
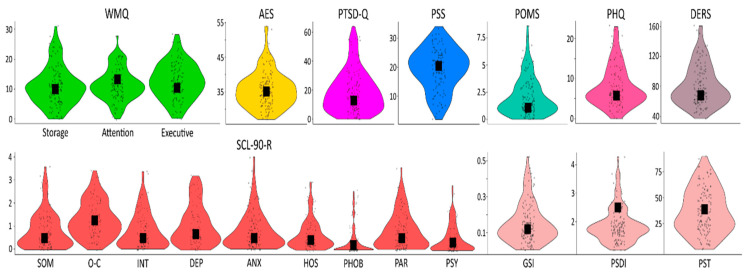
Violin plots representing the scores distribution for each scale and subscales of the eight questionnaires used in the study. Black squares indicate median scores.

Descriptive statistics and reliability index (Cronbach’s α) of our questionnaires are reported in Figure 4 and Table 1.

### 4.3. Relationship between Questionnaires and Behavioral Task Performance

#### 4.3.1. Effects on WM Performance

**Figure 5:**
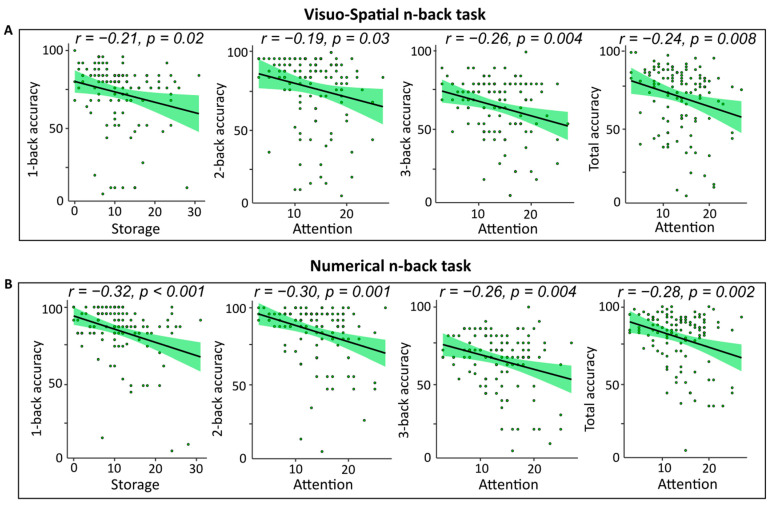
(**A**) Scatterplots of correlation between Storage and Attention subscales of the WMQ questionnaire and visuospatial n-back accuracies. (**B**) Scatterplots of correlation between Storage and Attention subscales of the WMQ questionnaire and numerical n-back accuracies.

As far as concern the relationship between scores from the Working Memory Questionnaire [20] and n-back performance, both in the case of VS and N blocks, Storage dimension (VS: β = −0.211, t118 = −2.3, *p* = 0.021; N: β = −0.328, t118 = −3.7, *p* < 0.001) was the only significant regressor to enter in a model explaining respectively 4.5% (F1,118 = 5.4, *p* = 0.021) and 10.8% (F1,118 = 14.2, *p* < 0.001) of the variance in the accuracy performance to 1-back trials. Negative zero-order correlations indicated that the larger the Storage scores to WMQ, the lower the accuracy to 1-back trials (see Figure 5A,B).

Similarly, in both VS and N blocks, accuracy to 2- and 3-back trials, as well as the total accuracy scores, were all significantly predicted by the Attention subscale of the WMQ (2-backVS: β = −0.198, t118 = −2.1, *p* = 0.03; 3-backVS: β = −0.26, t118 = −2.9, *p* = 0.004; TotalAccVS: β = −0.241, t118 = −2.6, *p* = 0.008; 2-backN: β = −0.303, t118 = −3.4, *p* < 0.001; 3-backN: β = −0.263, t118 = −2.9, *p* = 0.004; TotalAccN: β = −0.281, t118 = −3.5, *p* < 0.001). In these cases, Attention scores explained 3.1% (F1,118 = 4.8, *p* = 0.03), 6.8% (F1,118 = 8.5, *p* = 0.004), and 5.8% (F1,118 = 7.2, *p* = 0.008) of the variance in the VS block, respectively, while in the N block, they explained the 9.2% (F1,118 = 11.9, *p* < 0.001), 6.9% (F1,118 = 8.7, *p* = 0.004), and 9.9% (F1,118 = 12.9, *p* < 0.001) of the total variance. Also, in this case, zero-order correlations were all negative and pointed out that the larger the scores to the Attention subscale, the lower the n-back accuracies (see Figure 5A,B).

Regarding the questionnaires we used to evaluate mood, it emerged that POMS scores did not predict performance at any level of the n-back task. In the same vein, no significant results were found when AES, COVID-19 PTSD, PSS, PHQ-9, and DERS were used as regressors in our models.

**Figure 6:**
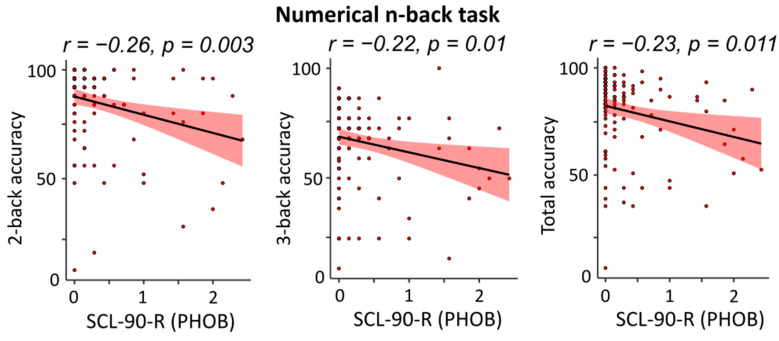
Scatterplots of correlation between the Phobic Anxiety subscale of the SCL-90-R questionnaire and numerical n-back accuracies.

In contrast, when subscales of the SCL-90-R were considered, the Phobic Anxiety dimension significantly predicted the accuracies of the numerical n-back task. In particular, PHOB explained respectively 7.2% (F1,118 = 7.1, *p* = 0.003), 5.1% (F1,118 = 6.3, *p* = 0.013), and 5.4% (F1,118 = 6.6, *p* = 0.011) of the variance in the performance of 2-back (β = −0.267, t118 = −3.0, *p* = 0.003), 3-back (β = −0.227, t118 = −2.5, *p* = 0.013), and total accuracies (β = −0.232, t118 = −2.5, *p* = 0.011) in this block. Negative zero-order correlations indicated that the larger the Storage scores to the PHOB sub-scale, the lower the accuracy in the numerical n-back (see Figure 6).

Please note that no relationship between our dependent variables and age/sex was found when these were considered as further independent variables in our stepwise regressions.

#### 4.3.2. Effects on Apathy Levels

**Figure 7:**
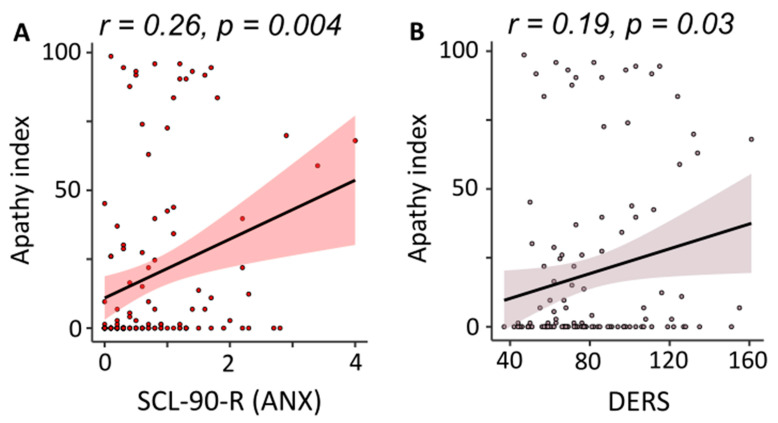
(**A**) Scatterplot of correlation between scores from the Anxiety subscale of the SCL-90-R questionnaire and Apathy index in the n-back task. (**B**) Scatterplot of correlation between scores from DERS questionnaire and Apathy index in the n-back task.

Regarding the analyses relative to the apathy, we found that Anxiety subscale of the SCL-90-R (β = 0.264, t118 = 2.9, *p* = 0.004) and DERS (β = 0.19, t118 = 2.1, *p* = 0.03) were the only significant regressor enter in models explaining respectively 7% (F1,118 = 8.8, *p* = 0.004), and 3.6% (F1,118 = 4.4, *p* = 0.03) of the total variance in the Apathy index during the whole n-back task. More specifically, zero-order correlations pointed out that participants with the highest anxiety and emotional dysregulation levels decided to skip trials more frequently (see Figure 7). Also, in this case, no relationship was found with age and gender of our sample.

## 5. Discussion

The present study examined the influence of mood on working memory capacity and apathy. We tried to evaluate this influence using a battery of eight questionnaires, a behavioral n-back task [36], and a measure for apathy.

As a first result, we found that during the n-back task, participants performed better in the Numerical Condition (N) than in the VisuoSpatial (VS) one. In our experimental design, participants always completed the VS-block before the N-block. Therefore, this difference could simply reflect a general practice effect or higher familiarization with the task. This is in line with a study showing that two sessions of n-back are sufficient to elicit general improvement in performance [40]. Another possibility would be that the different results between numerical and visuospatial n-back tasks are due to differences in the stimuli used. Indeed, numerical stimuli are generally more familiar and easier to encode than visuospatial ones [41]. For example, everyone is familiar with the numbers 1–10, but not everyone is familiar with all possible visuospatial configurations.

Notwithstanding these basic interpretations, to our knowledge, studies that systematically compare VS and N n-back conditions are currently lacking; therefore, it is not possible to fully exclude that this effect could be genuinely related to some distinctions in the WM mechanisms underlying the two different modalities of task execution [42]. For example, numerical working memory might be more efficient than visuospatial working memory because numbers can be represented in a more abstract way than visuospatial information, which requires a more complex and concrete representation [43]. In this regard, studies about neural correlates of VS-WM showed that performance to the VS n-back using grid task depends on concomitant activations of both frontal regions associated with executive control and more posterior regions associated with storing information [42,44]. Conversely, numerical working memory is more closely linked to language processing [45,46] and to posterior parietal cortical regions [42]. This is because numbers are often used in language, whereas visuospatial information is not. Additionally, another work [47] proposed that WM had a capacity limit that did not apply to individual objects per se but rather to the bindings connecting items to one another or items to context. For instance, in an experiment with digit span, it is assumed that capacity is not restricted to digits. This implies that during the majority of the trial, all nine out of ten digits are likely to have a high level of memory activation. Perhaps what is significant are the correspondences between items and serial locations, as well as between several items constituting a chunk, and the knowledge that certain numbers were included in the most recent list. Similar to this, it is crucial to know that specific positions were present in the most recent presentation while conducting a visuospatial comparison experiment. Bindings inside an item and bindings between an object and a physical place were distinguished by two authors in particular [48]. They discovered that people could remember many characteristics of an item at once, tied together. The capacity to compare two arrays, for instance, was no poorer when an item could vary in either color or orientation than when it could only change in one of those properties. In contrast, individuals were only able to remember roughly four of the location-specific items in an array. Based on these considerations, even within the constraints of our experimental design, our findings may help to shed insight into the mechanisms behind visuospatial and verbal/numerical domains on WM performance.

As a second result, we reported that, independently of the n-back modality, participants had a better performance in the 2-back as compared to 1-back, though decreasing their accuracies as a function of the n-back difficulty, with lower scores in the 3-back. The lower performance of participants in the 3-back condition is in line with classic results from n-back studies [42]. However, unexpectedly and at odds with previous results [49,50,51,52], we found a slightly better performance to 2-back rather than 1-back. We argue that this is mainly due to the differences in the rewards that were provided as feedback for our participants. Indeed, in the 2-back, we used a greater virtual reward (10$) than in the 1-back (1$). This could have led participants to experience more motivation and commitment through this part of the task and, therefore, perform better in the 2-back condition compared to 1-back. Interestingly, this finding is consistent with a series of studies showing that reward motivation and expectancy processed in the ventrolateral PFC can play a critical role in driving WM performance [53,54,55,56].

Notably, apathy level measured through the number of skipped trials did not modulate behavioral performance. This is in contrast to a study [57] where authors investigated the impact of apathy on cognitive performance in the healthy elderly. In particular, they found that general apathy and the sub-dimension of intellectual curiosity were associated with lower cognitive performance. However, in line with our results, Onyike and colleagues [58] did not find any significant relationship between apathy and memory. These contrasting results could be explained by the different measures and populations used in these studies. For example, while Motoya-Murillo et al. [57] and Onyike and colleagues [58] used questionnaires aiming at different dimensions of apathy, in this study, we measured the number of skipped trials, which, perhaps, could not adequately catch this theoretical construct. Another possible interpretation of these findings is that in a young population, reward expectation is more important than apathy level in predicting changes in WM performance.

Regarding the main aim of our study, we found two important negative results. As a first point, mood assessed by POMS [27], PHQ-9 [29], and DERS [31] did not show significant effects on n-back performance and, thus, on WM capacity. This result, contrary to a series of studies suggesting positive [4,5,6] or rather negative [7,8] effects of mood on WM capacity [9,10], seems rather to confirm observations of no evident effects of negative mood, neither on visuospatial tasks nor on verbal ones with high demand executive [11,12,13,14]. What emerges from our study is that mood has a rather limited effect on cognition. On the basis of this result, a further hypothesis could be made, namely that the n-back [36] can be considered as a biomarker of the depressive state. Indeed, previous observations reported that performance in the n-back task is compromised in patients with depressive disorder [59,60,61,62,63]. In contrast, in healthy subjects, results from the present and previous studies reported that performance on the n-back task is not impaired by negative mood [11,12,13,14]. On this ground, it could be hypothesized that a non-compromised n-back [36] indicates the absence of a real state of depression or its presence in a mild form. Therefore, even in legal contexts, this task could be used as a biomarker to exclude or confirm the presence of a depressive state.

Analyzing the effect of stress on task performance, we found no significant effects, so our findings do not support the data found by Schoofs and colleagues [16], who conducted a study evaluating the effects of stress on working memory and found that stressed subjects had significantly slower reaction times and provided fewer correct answers than the control group. Conversely, our findings are most similar to those of Porcelli et al. [17], who discovered a little effect of stress on WM performance, but we cannot corroborate them because we did not detect any effect.

Another important negative result is the lack of correspondence between the AES scores [22] and the value of apathy obtained from the n-back task. Several variables might account for this. First, due to social desirability, the answers to self-report surveys may not be totally accurate [64]. Second, it is possible to speculate that there are several levels of apathy, some of which are below the threshold and for which there is no complete awareness. As a result, they would only be discovered by the task rather than the questionnaire. This is because the subjects answer the self-report questionnaires based on what they are aware of and consider the possible consequences of what they say, while they are unaware of what is being investigated during the task so that social desirability will affect participants’ behavior less.

By contrast, good correspondence between questionnaire scores and behavioral performance was found in the case of the WMQ. Indeed, linear regressions showed that scores from the Attention subscale could be considered a good predictor of the most difficult level of the n-back task, both in the visuospatial and in the numerical domain. Storage subscale instead predicted performance to the easiest step of this task (1-back). These findings might be considered in light of the differing cognitive demands at the various levels of n-back. During the 1-back condition, participants had to compare the current trial with the previous one so that the task only dealt with the memory processes of encoding and retrieval at their most basic level. Conversely, the 2- or 3-back conditions also involve higher-order working memory processes such as attention, inhibition, and cognitive control for the maintenance of temporal position information [65]. In this regard, different neuroimaging studies showed that subregions in the prefrontal, parietal, and visual association areas were load-dependently activated during the performance of the n-back tasks. In particular, simple storage and maintenance (1-back) are associated with lower levels of activity in a few brain locations, including the inferior parietal and dorsolateral prefrontal cortex [45,50,51,52]. During the 2-back task, however, a consistent, widespread activation was found in the dorsolateral/ventral prefrontal cortex, as well as the superior and inferior parietal lobule, occipital visual association regions, anterior and posterior cingulate areas, and the insula [50,52]. Interestingly, brain activity unique to information manipulation in WM (2-back > 1-back comparison) resulted in increased and equal dorsolateral prefrontal cortex and anterior cingulate activation [52].

Interestingly, psychiatric symptoms seem to have a sort of influence on WM capacity, especially in the digit domain. In fact, the SCL-90-R’s phobic anxiety dimension [33] had a significant effect on performance at numerical 3-back, numerical 2-back, and total numerical block with a negative regression coefficient, indicating that a higher level of phobic anxiety would affect working memory capacity, resulting in impaired task performance. These findings are corroborated in part by Yang et al. ‘s study [10], which examined the influence of emotional states on visual working memory in trait anxiety and found that emotionality mixed with anxiety has an effect on WM that is dependent on both the kind of activity and the emotional state. Based on this, one may argue that, at least in part, anxiety impairs working memory capacity.

Finally, emotional dysregulation as measured by DERS [31] and the Anxiety component (SCL-90-R) influenced apathy level. We discovered that the higher these scores, the greater the apathy index. To our knowledge, this novel finding has not been previously observed in the literature. Therefore, it would be crucial to conduct more research to investigate this association, especially given the increased interest in isolated apathy in recent years, i.e., in the absence of mood disorders. For example, Pardini et al. [18] found that approximately 1.45% of healthy subjects had significant levels of apathy; these participants demonstrated less behavioral activation than controls, but no differences in fatigue, depression, self-efficacy, behavioral inhibition, and perceived social skills were observed. However, because the authors did not assess the participants’ mood, it is unknown if individuals in the “apathy” group had a lower mood as well.

## 6. Conclusions

In summary, our study revealed mixed findings, with partial support for our initial hypothesis, as no mood-related differences were observed in working memory task performance. However, we did find a strong correlation between WMQ scores and behavioral working memory performance in both visuospatial and numerical domains. Furthermore, the SCL-90-R’s phobic anxiety dimension negatively impacted numerical n-back task performance, and the same Anxiety component, along with emotional dysregulation, influenced apathy levels, showing that higher scores were associated with greater apathy index.

## 7. Limitations and Future Prospects

Here, we wish to consider some limitations of the present study. First, both the questionnaire and the experiment were administered online. Therefore, notwithstanding that task instructions were carefully provided via individual online meetings, we had no direct control over the presence of external distracting events or potential confounding variables during task performance. For this reason, a possible future evolution of such a study could be to repeat the measurements in a controlled condition in the laboratory. A second limitation regards the use of self-report questionnaires and the measure of apathy detected by the task. Indeed, we do not have the certainty that participants skipped the trials due to a lack of motivation. At some point, they may have started jumping because, given the length of the task, they wanted to finish as soon as possible; in fact, we did not find consistency between the results of the AES [22] and the level of apathy that emerged from the task; although another explanation for this could be the one assumed in the discussion. A future perspective to improve the study from this point of view could be to create a behavioral task that detects apathy more precisely.

Finally, we would like to emphasize both the relevance of the present type of real-world data and the importance of learning how to deal with the inherent limitations of a naturalistic setup when moving out of the laboratory.

## Figures and Tables

**Table 1:** Descriptive statistics and reliability analysis of questionnaires used in the study.

Descriptive Statistics		WMQ						SCL-90														
	Age	Storage	Attention	Executive	AES	PTSD-Q	PSS	SOM	O-C	INT	DEP	ANX	HOS	PHOB	PAR	PSY	GSI	PSDI	PST	POMS	PHQ	DERS
Mean	27.8	11.1	13.6	11.5	35.6	19.1	19.3	0.806	1.30	0.782	1.06	0.789	0.619	0.335	0.779	0.464	0.150	1.88	37.2	2.06	7.80	80.3
Median	24.0	10.0	14.0	11.0	35.0	16.0	20.5	0.583	1.20	0.556	0.846	0.550	0.333	0.143	0.583	0.200	0.124	1.76	34.0	1.47	6.00	73.0
S.D.	10.5	6.49	5.16	5.33	5.22	14.4	7.26	0.757	0.772	0.756	0.855	0.783	0.593	0.570	0.758	0.563	0.110	0.612	21.8	1.75	4.99	26.7
95% C.I. SUP	25.9	9.88	12.7	10.6	34.6	16.5	18.0	0.669	1.16	0.646	0.905	0.648	0.512	0.231	0.642	0.362	0.130	1.77	33.3	1.74	6.90	75.5
95% C.I. INF	29.7	12.2	14.5	12.5	36.5	21.7	20.6	0.943	1.44	0.919	1.21	0.931	0.727	0.438	0.916	0.566	0.170	1.99	41.1	2.37	8.70	85.2
Min	18.0	0.0	3.0	0.0	27.0	0.0	2.0	0.00	0.00	0.00	0.00	0.00	0.00	0.00	0.00	0.00	0.00333	1.00	0.0	0.00	0.0	37.0
Max	60.0	31.0	27.0	28.0	54.0	64.0	34.0	3.50	3.40	3.33	3.15	4.00	2.83	2.43	3.50	2.70	0.523	4.27	90.0	8.60	23.0	161.0
Cronbach’s α	-	0.92			0.70	0.93	0.89	0.97												0.94	0.85	0.95

## Data Availability

The data presented in this study are available on request from the corresponding author. The data are not publicly available due to the sensitive information contained.
